# Long-term segregation in mental health hospitals: qualitative study of its impact and human rights implications

**DOI:** 10.1192/bjo.2026.10996

**Published:** 2026-03-27

**Authors:** Kathryn Fradley, Alina Haines-Delmont

**Affiliations:** School of Nursing and Public Health, Manchester Metropolitan University, UK

**Keywords:** Mental health services, human rights, qualitative research, intellectual disability, autistic spectrum disorders

## Abstract

**Background:**

Long-term segregation (LTS) is used in mental health hospitals in England to manage individuals perceived to pose a sustained risk of harm to others. Increasing evidence indicates that LTS causes significant psychological and physical harm and may breach international human rights standards. The HOPE(S) programme (2022–2025) was introduced nationally to reduce, and ultimately end, the use of LTS for autistic people, individuals with learning disabilities and children and young people.

**Aims:**

To explore the experiences of LTS from different perspectives and to examine its impact through a human rights lens.

**Method:**

Qualitative data were collected from 73 participants, including people with lived experience of LTS, family members, HOPE(S) practitioners, clinical staff, commissioners and regulators. Reflexive thematic analysis was conducted as part of a wider, mixed-methods evaluation of the HOPE(S) programme.

**Results:**

LTS was described by most participants as harmful, dehumanising and lacking in therapeutic value. Four interrelated themes emerged: (a) dehumanisation and erosion of personhood; (b) safeguarding and systemic failure; (c) psychological and relational harm; and (d) loss of hope and systemic inertia. These experiences reflected breaches of the Convention on the Rights of Persons with Disabilities, and of the Convention on the Rights of the Child.

**Conclusions:**

LTS is not a therapeutic intervention and is associated with profound psychological harm and human rights violations. Systemic reform and implementation of rights-based, trauma-informed alternatives, such as the HOPE(S) model, are urgently required to safeguard well-being and dignity in mental healthcare.

## Long-term segregation under UK law

There is increasing concern that long-term segregation (LTS) within mental health settings involves practices that are both harmful and inconsistent with human rights obligations, particularly the right to protection from inhumane or degrading treatment.^1–3^ In the UK, LTS is defined in the Mental Health Act 1983: Code of Practice^4^ as ‘…prolonged isolation of an individual who is perceived to pose a sustained risk of harm to others, preventing social interaction with peers, while maintaining limited staff contact’.^4^ Although sometimes described in policy literature as ‘solitary confinement’, this is not a formal legal term. The current framework does not specify a maximum duration for LTS; instead, continuation requires regular review by a multidisciplinary team to justify ongoing segregation and consider less restrictive alternatives. Empirical reporting on typical durations of LTS is limited, possibility reflecting the hidden and complex nature of the practice.^5,6^ Regulatory reviews have documented adults in LTS for up to 9.5 years and children or young persons for up to 2.4 years, with secondary reports suggestions cases of up to approximately 13 years.^6,7^

## Human rights concerns and ethical implications of LTS

Recent independent reviews have concluded that all forms of solitary confinement, including LTS, lack therapeutic benefit and may cause lasting psychological harm.^3^ These findings reinforce long-lasting concerns raised by advocacy organisations such as Mencap^2^ and the National Autistic Society^8^ regarding the use of LTS for children and young people, autistic adults and adults with learning disabilities. The practice has been described as a ‘never event’, permissible only in exceptional circumstances and requiring rigorous human rights safeguards.^3^ Although UK policy frames LTS as a measure of last resort,^4^ recent reports suggest that it is often prolonged and disproportionately used on children, young people, autistic adults and adults with learning disabilities.^2,3,6–8^ Emerging evidence suggests that LTS in its current form falls short of the UN Convention on the Rights of the Child (CRC) and the UN Convention on the Rights of Persons with Disabilities (CRPD).^9–11^ First, LTS may contravene CRC Article 37, which prohibits torture and other cruel, inhuman or degrading treatment or punishment of children and limits deprivation of liberty to lawful, non-arbitrary use only as a measure of last resort. Similarly, under CRPD, LTS may raise concerns in relation to Article 14 (liberty and security of the person), Article 15 (freedom from torture or cruel, inhuman or degrading treatment or punishment) and Article 17 (protection of physical and mental integrity), particularly where isolation is prolonged, unnecessary or imposed without independent oversight of the deprivation of liberty. These concerns are reflected in the upcoming UK Mental Health Bill (2025),^12^ which calls for strengthened protection of the rights and well-being of people detained under the Mental Health Act. Whereas some commentators have questioned whether the evidence base on the harms of LTS is sufficient,^13^ this position has been criticised for overlooking the depth and consistency of testimony from people directly affected^1^ in research exploring the impact of LTS. To date, few empirical studies have examined the lived experience of LTS or its impact through a human rights framework. Understanding LTS as a potential site of rights violations provides a critical lens through which to evaluate its ethical and clinical legitimacy, and to inform future policy and practice.

## Current study

The present study draws on qualitative data collected as part of the national evaluation of the HOPE(S) programme,^14^ introduced in England to reduce, and ultimately end, the use of LTS for autistic people, individuals with learning disabilities and children and young people. The programme adopted the rights-based HOPE(S) model:^15^ this encourages teams to harness the system through key attachments and partnerships; create opportunities for positive behaviours, meaningful and physical activities; identify protective and preventative risk and clinical management strategies; and build interventions to enhance the coping skills of both staff and people in services. In addition to supporting transitions out of LTS, the HOPE(S) programme – henceforth referred to as HOPE(S) – aimed to improve care quality and well-being for those currently subjected to segregation. Individuals who have experienced LTS at first hand offer unique insights into its psychological, emotional and social consequences. Alongside them, family members provide a vital perspective on how segregation affects relationships, trust in services and long-term well-being. The accounts of clinical staff, commissioners, regulators and HOPE(S) practitioners are also essential because they reveal the emotional, ethical and systemic costs of segregation, even among those tasked with sustaining or challenging such practices. Although only a small number of participants had direct lived experience, integration of these diverse perspectives enables a fuller understanding of how LTS is enacted, rationalised and resisted within institutional systems. By situating these accounts within a human rights framework, the study seeks to illuminate the profound ethical, psychological and systemic implications of LTS and to contribute to the evidence base for rights-based alternatives in mental healthcare.

## Method

### Design and context

This qualitative study formed part of a national mixed-methods evaluation of the HOPE(S) programme (2022–2025),^14^ a rights-based intervention implemented in England to reduce, and ultimately end, the use of LTS for autistic people, individuals with learning disabilities and children and young people. The programme was delivered across 68 mental health hospitals within 40 National Health Service (NHS) commissioned services. Trained HOPE(S) practitioners supported individuals in LTS and their clinical teams and partnered with Respond, a trauma-informed therapeutic service providing specialist support for families.

### Participants and recruitment

Seventy-three participants contributed to the qualitative strand, representing a range of perspectives: people with lived experience of LTS (*n* = 4), family members (*n* = 11), HOPE(S) practitioners (*n* = 10), clinical staff, professionals working for non-profit organisations, commissioners and regulators (*n* = 43) and Respond practitioners (*n* = 5). Although only a small number had direct lived experience, the inclusion of these voices was considered essential to centring the human impact of LTS. The perspectives of family members and professionals provided important contextual insight into the social, relational and systemic consequences of segregation.

Many participants were recruited through HOPE(S) sites using volunteer sampling: professionals, family members and individuals with lived experience decided to take part once informed of the study. This is the most appropriate approach for engaging individuals with specific, often hard-to-reach experiences. The research team were supported by HOPE(S) intervention staff, the project steering group and national HOPE(S) stakeholder events to identify eligible participants. Interested stakeholders were invited to contact the research team directly. Additionally, some participants were recruited following direct contact with researchers at national networking events. Written informed consent was obtained from all participants. To protect anonymity, all participants were assigned numerical identifiers.

### Data collection

Data were collected between July 2023 and January 2025 through semi-structured interviews and focus groups, guided by topic schedules tailored to participant role and experience. (This study followed the ‘COnsolidated criteria for REporting Qualitative research (COREQ)’ checklist, a full copy of which can be found in the Supplementary Material, available at https://doi.org/10.1192/bjo.2026.10996.) Two waves of data collection were conducted during this period.

The first wave (between July 2023 and January 2024) primarily comprised in-depth interviews with family members, intervention practitioners and a small number of clinical staff, with the aim of exploring experiences and views on the implementation of HOPE(S). When deemed more appropriate, alternative formats (focus groups or self-completion questionnaires/written interviews) were offered on a small number of occasions. Findings from the first wave informed an interim report for the HOPE(S) evaluation, which indicated the need for additional recruitment and amended questions about the impact of LTS, the future of HOPE(S) and implications for families, policy and practice, particularly among clinical staff, managers, commissioners and regulators.

The second wave (between April 2024 and January 2025) therefore used a combination of interviews and focus groups, with a greater emphasis on group-based methods, to support broader recruitment and capture a wider range of stakeholder perspectives.

Overall, 73 individuals participated in this study. Three family members and two Respond practitioners asked either to participate more than once or to join another form of data collection (i.e. subsequent focus groups), because they felt they had more information to share. These individuals were counted only once when reporting total participant numbers (*N* = 73).

A total of 50 interviews were conducted with HOPE(S) practitioners (*n* = 10), family members (*n* = 11), clinical staff (*n* = 19), other professionals (*n* = 6) and Respond practitioners (*n* = 4). Two family members completed written responses due to accessibility needs.

A total of nine focus groups were conducted:

- four focus groups consisting of only clinical staff (*n* = 10);
- one focus group consisting of Respond practitioners (*n* = 3);
- one focus group consisting of commissioners (*n* = 4);
- one focus group consisting of family members (*n* = 2);
- one focus group consisting of individuals with lived experience (*n* = 4);
- one focus group consisting of four clinical staff and a Respond practitioner.

Most interviews and focus groups were conducted online via Microsoft Teams and recorded with participants’ consent. One focus group comprising individuals with lived experience was held face to face and audio-recorded only. Support workers were present in sessions involving individuals with lived experience or families, to safeguard well-being and provide post-interview support. Researchers maintained reflexive field notes to capture contextual observations and emotional responses.

The findings were co-produced with someone with lived experience of restrictive practices, including LTS, as well as a family member whose loved one had extensive experience of LTS.

### Ethics

The authors assert that all procedures contributing to this work comply with the ethical standards of the relevant national and institutional committees on human experimentation, and with the Helsinki Declaration of 1975 as revised in 2013. All procedures involving human subjects/patients were approved by Manchester Metropolitan University (EthOS ID: 46401), the South-East Scotland Research Ethics Committee (10 May 2023) and the Health Research Authority (16 May 2023; IRAS: 319279).

### Analysis

The findings presented here were analysed as part of a wider study to evaluate the HOPE(S) programme.^14^ Data were transcribed verbatim and analysed in NVivo 14 for Windows (Lumivero, Denver, Colorado, USA; https://lumivero.com/products/nvivo/) using reflexive thematic analysis.^16,17^ Due to the large volume of qualitative data, initial coding was undertaken by one member of the research team (K.F.). To enhance interpretive rigour and mitigate potential biases, several strategies were employed. These included peer-debriefing, involving discussion of codes, themes and interpretations, as well as random double-coding checks (moderation) with the co-author (A.H.-D.) to identify and resolve discrepancies in interpretation. The co-author was familiar with the data because they either conducted or supervised data collection throughout and had previously conducted in-depth analysis for other research questions. Additionally, sense-checking and co-production were used to further validate the interpretations.

Data saturation was considered reached when interviews no longer yielded new information or insights relevant to the research questions. Although data saturation was identified during data collection, interviews continued in order to ensure thoroughness, and to include all participants who expressed interest in being interviewed. The additional interviews confirmed the existing themes and did not contribute novel information, thereby reinforcing the fact that data saturation had been achieved.

The analysis was inductive, focusing on how participants conceptualised the psychological, relational and systemic impacts of LTS. Co-production was integral to the analytic process: findings were reviewed and refined collaboratively with a person with lived experience of restrictive practices and a family member of someone who had experienced LTS. This reflexive stance positioned the research team as co-witnesses, acknowledging the emotional and ethical labour involved in listening to accounts of trauma and degradation.^18,19^ The authors were particularly driven to strengthen the currently underrepresented voices of people directly impacted by LTS within the academic sphere, thereby contributing to a shift in existing power imbalances. However, given that the authors had no personal experience of LTS, they remained conscious that their professional backgrounds, assumptions and relative positions of power had the potential to influence their interpretation of the qualitative data, especially regarding how LTS is understood and represented.

Reflective discussion and co-production therefore informed analytic discussions throughout the process. This was considered essential to challenging these interpretive biases, and to addressing existing power imbalances within academic understandings of LTS. In practice, this involved questioning early analytic framings that risked overemphasising organisational processes or clinical rationales, and re-centring the analysis on lived experiences of loss, degradation and relational harm as articulated by contributors with lived experience. Reflexive discussions also informed decisions about language, ensuring that the terms used to describe LTS reflected participants’ own characterisations rather than institutional terminology (especially around ‘risk’). Through this reflexive and co-productive process, preliminary themes were revised to foreground the devastating impacts of LTS as articulated by those with direct experience.

Contributors with lived experience described a profound sense of desperation to be heard, and strongly characterised LTS as a degrading, inhumane and emotionally – and at times physically – destructive practice. The authors believe that incorporation of these perspectives ensured that the final themes more accurately reflected the priorities, language and concerns of those most directly affected by LTS. In doing so, this approach also sought to strengthen and amplify voices that remain underrepresented within the academic literature on LTS.

## Results

Across stakeholder groups, participants consistently described LTS as profoundly harmful and lacking any therapeutic value. People with lived experience, their families and professionals portrayed LTS as a practice that isolates, dehumanises and ultimately destroys the possibility of recovery. The majority of participants described the impact of LTS as harmful. Individuals with lived experience described it as ‘devastating and horrible’ and said that it ‘makes you more angry and more distressed’ (participant 64, lived experience). Their environment was perceived as traumatic, overwhelming and anxiety-provoking. Family members and many professionals corroborated these experiences, describing how LTS led to severe deterioration in both physical and mental health. The poor quality of life associated with segregation caused desperation and regret among families, some of whom questioned whether hospital admission had protected or harmed their loved one:

‘So, she’s been officially in long term segregation since [date], not walking, not talking and she’s now not eating hardly anything in about five or six weeks. She’s lost so much weight’ (participant 20, family member).

‘Their quality of life, I would say, is challenged. Limited access to personal items, limited access to family, limited or no access to fresh air’ (participant 22, commissioner).

‘At one point I said, please just let her die because it feels like you are just killing her anyway’ (participant 18, family member).

Building on these shared experiences, the analysis identified four interrelated themes that illustrate the psychological, relational and systemic consequences of LTS:

- stripped of identity: the loss of self in isolation (dehumanisation and erosion of personhood);
- the hatch: a door to abuse and neglect (safeguarding and systemic failure);
- living in fear: distress, trauma and family despair (psychological and relational harm);
- broken spirit: the harm of therapeutic abandonment (loss of hope and systemic inertia).

**Fig. 1:**
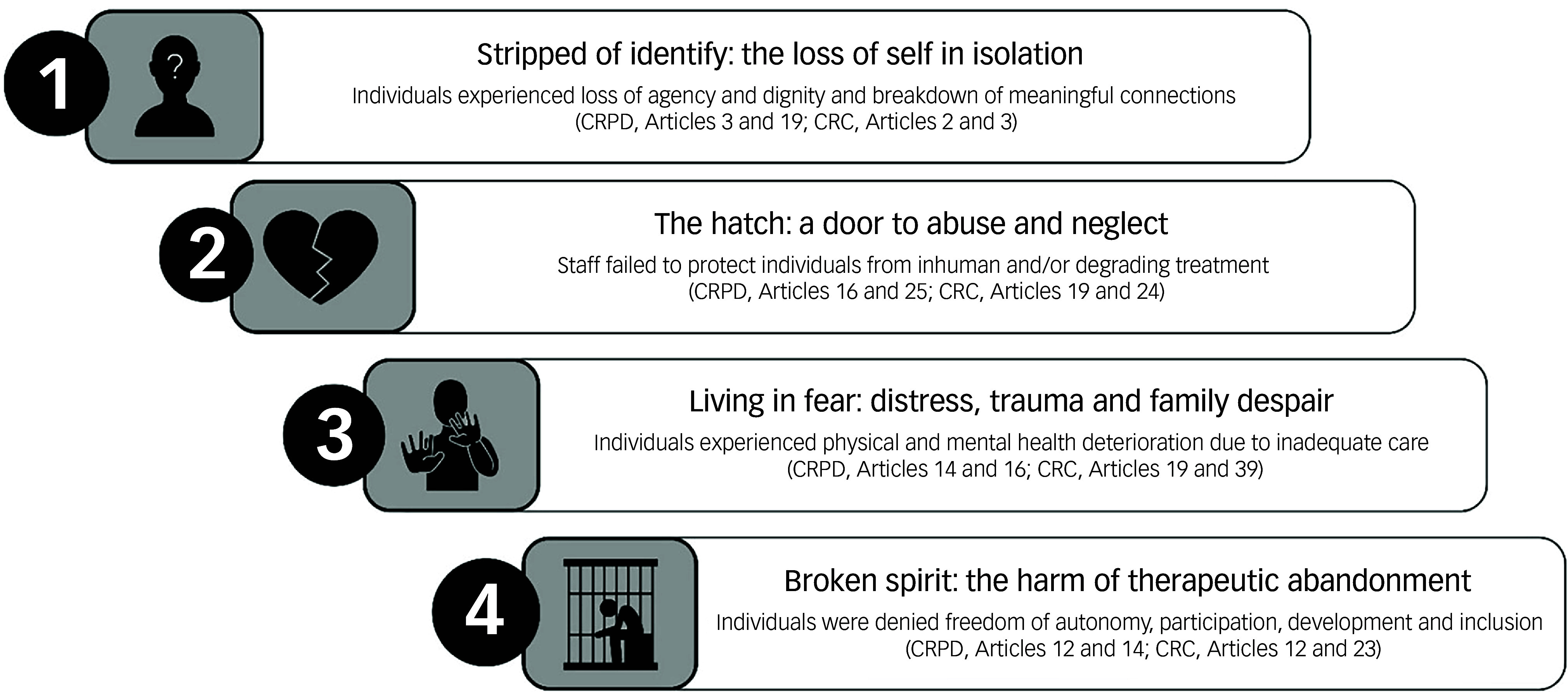
Long-term segregation: a practice that hurts more than it helps. CRPD, Convention on the Rights of Persons with Disabilities; CRC, Convention on the Rights of the Child.

Together, these themes illuminate how LTS undermines dignity, autonomy and protection, principles enshrined in both CRPD and CRC, demonstrating that LTS functions not as a therapeutic measure but as a form of sustained deprivation and harm. ‘Safeguarding’ here refers to the duty to protect people from abuse, neglect and harm, and to take action to promote their safety, well-being and rights while under care.^20^ Figure 1 summarises the relationship between each theme and corresponding rights violations under CRPD and CRC.

### Stripped of identify: the loss of self in isolation (dehumanisation and erosion of personhood)

There was consensus among participants that individuals in LTS were often dehumanised, leading to an erosion of people’s sense of self. This reflects how restrictive institutional practices systematically strip individuals of their autonomy, identity and dignity. These accounts reflect how LTS functions as more than just a physical separation, because it actively strips away personhood through enforced isolation, loss of agency and the reinforcement of ‘otherness’:

‘…it dehumanises my gorgeous son’ (participant 40, family member).

‘…but we almost sort of dehumanise the person’ (participant 41, commissioner).

‘There’s a real dehumanisation when we get into long-term segregation and the othering’ (participant 53, clinical staff).

‘…I think [professionals] would act really differently if they could just imagine it was their family or it was them that was locked up every day and restrained every day… they would think differently’ (participant 76, family member).

Several described LTS as ‘serving a prison sentence’ (participant 5, HOPE(S) practitioner), where individuals were viewed as criminals rather than patients. Over time this framing was internalised, with individuals perceiving themselves as dangerous or undeserving of care:

‘He went in as a happy go-lucky boy with a catatonic breakdown and he’s now classified as a very dangerous person, and he thinks he’s dangerous. He thinks he needs an Ironman suit to keep his arms and legs safe. He is completely destroyed as a human being’ (participant 40, family member).

‘And the problem we have in managing her now is she thinks she deserves to be punished and to suffer and does not want to come out of segregation particularly’ (participant 30, clinical staff).

‘The staff are restricting someone’s use of something that they love and that helps regulate them but actually they have removed that and it feels like that’s been removed as a punishment or consequences of behaviour, challenging behaviour rather than actually that’s the thing that helps regulate that challenging behaviour’ (participant 12, Respond practitioner).

Participants felt that individuals in LTS lost their identity and sense of self as they internalised this ‘risk’ narrative. Many believed that individuals came to perceive themselves as dangerous criminals, just as clinical staff do. This distorted self-image led to fear of harming others and a belief that they deserved to be in isolation. As a result, some individuals chose to self-isolate, believing it was a way to protect others from themselves or, in some cases, to protect themselves from others. Family members were devastated at the thought of their loved one losing their identity and self-worth. HOPE(S) practitioners also observed these patterns, highlighting the damaging psychological impact of LTS and the urgent need for more compassionate, person-centred care.

Overall, participants described how LTS extinguished individuality and dignity, leading to self-stigma and internalised fear. These experiences represent breaches of fundamental rights to dignity, autonomy and inclusion (CRPD Articles 3, 19; CRC Articles 2, 3).

### The hatch: a door to abuse and neglect (safeguarding and systemic failure)

Safeguarding concerns were widespread. This included neglect, as well as financial, physical and emotional abuse, often perpetrated by staff. Participants across all groups recounted or witnessed neglect and abuse: physical, emotional and institutional. Several described in detail specific safeguarding incidences where individuals were deprived of privacy, dignity and even basic care. These were described as deeply distressing and traumatic, underscoring the urgent need for stronger protections and accountability within LTS settings:

‘…examples of not just being able to go to the toilet on your own but having to pass out your waste through a hatch. That’s real. That happened’ (participant 53, clinical staff).

‘He was locked behind a door and the only interaction was through a hatch and still abuse, psychological and physical, took place’ (participant 32, clinical staff).

‘… I would raise a safeguard to get them to check on his welfare. There were numerous times we’d call the police as well for a welfare check but they always turned around and said that he was in hospital so he was safe’ (participant 16, family member).

‘It just brought back so much trauma from [the hospital] where he was literally fed through a hatch for months on end and just medicated and left in his own faeces’ (participant 17, family member).

‘There was about five safeguarding incidents from the service towards the person […] incidents where I felt that harm had been caused to her by staff actions’ (participant 3, HOPE(S) practitioner).

For individuals with experience, LTS also meant a lack of meaningful human connection, compassion and empathy from clinical staff, especially during times of severe distress. Many expressed a deep desire for someone to talk to during moments of distress yet felt unable to communicate this need. Staff were often seen to be unable to address this need, leading to further isolation, particularly as staff were the only people to which they had access. Individuals with lived experience also spoke of profound feelings of loss because they missed their families (including pets) and the meaningful activities that once brought them comfort, such as visiting the park or listening to music.

‘…it made me more wound up because you’re already feeling out of control, and I was already in that position because of other comments that were made, and I didn’t know how to deal with the stress so I’d take it out on myself. Because I couldn’t self-harm by cutting, which is what I used to do, I would then bang my head. The staff wanted to intervene but were actually told by their manager to just leave it and just sit there and basically watch…

… you will get a lot of comments which would then make you worse. I think I had one, she went on to be a manager which I wasn’t happy about. She told me that if I did want to die, I would take a stronger overdose’ (participant 64, lived experience).

Overall, these accounts reveal violations of the right to adequate care, to family life and to protection from abuse. These experiences represent breaches of fundamental rights to dignity, autonomy and inclusion (CRPD Articles 3, 19; CRC Articles 2, 3). Accounts of harm in LTS settings thus expose systemic failures to ensure safe, rights-based care, reinforcing the urgent need for more compassionate and rights-based approaches to care in LTS settings.

### Living in fear: distress, trauma and family despair (psychological and relational harm)

LTS was widely described as frightening and traumatising for individuals, and families lived in constant fear for their loved ones’ safety:

‘He was so frightened of being shut in there’ (participant 19, family member).

‘The amount of times when we thought he was going to be dead’ (participant 17, family member).

‘[staff] go home at night and they don’t have to worry about every time they get a private number on their call, it’s going to be the call to say their child is dead’ (participant 76, family member).

Family members expressed deep mistrust of services, shaped by previous neglect and lack of transparency. HOPE(S) practitioners corroborated this further because they often talked about the difficulties in gaining the trust of individuals whom they were supporting (in LTS).

Additionally, participants felt that individuals in LTS were traumatised and re-traumatised by the system, as well as by certain clinical staff. Many described the LTS environment as frightening, often triggering memories of past traumatic experiences. Re-traumatisation was particularly common during hospital transfers, especially when individuals were returned to settings where they had previously experienced neglect or abuse. These experiences also deeply affected family members, compounding their distress and sense of helplessness. There were attempts made by family members, HOPE(S) practitioners and even some clinical staff to stop planned hospital transfers, believing that the individual’s mental and physical health would probably deteriorate once moved. However, they were often unsuccessful:

‘And then they end up traumatised from being in hospital, then re-traumatised… it’s a never-ending circle’ (participant 22, commissioner).

‘We begged them. We said, “Please, we’ve all had such a horrific experience there, please don’t send her back”, but they did’ (participant 20, family member).

Overall, accounts of fear and trauma highlight the adverse impact of LTS. Participants described states of distress and desperation, often feeling powerless to intervene. Such accounts depict a cycle of institutional trauma that undermines recovery and trust. These experiences contravene rights to liberty and security (CRPD Article 14), and to freedom from mental violence and re-traumatisation (CRC Articles 19, 39).

### Broken spirit: the harm of therapeutic abandonment (loss of hope and systemic inertia)

The final theme captures the pervasive hopelessness described by individuals, families and professionals. Participants talked about how individuals in LTS were not only physically trapped in isolation, but also therapeutically and mentally stranded by clinical staff. They described a pervasive sense of hopelessness among individuals in LTS, who probably believed they were destined to remain in enforced isolation indefinitely without meaningful support, human connection or engagement in purposeful activities. This sense of helplessness was described as deeply affecting individuals’ mental well-being and further entrenched their distress. As a result, individuals in LTS later struggled to engage with the therapeutic interventions introduced by HOPE(S) practitioners, because they had lost faith in the possibility of change or recovery. Taken together, participants portrayed people in LTS as being ‘stuck’: physically confined and psychologically immobilised by institutional inertia:

‘I think for some service users they might feel hopeless and are not particularly willing or not particularly in a position where they feel able to be hopeful enough that somebody else could come in and could help them’ (participant 1, HOPE(S) practitioner).

‘I’ve certainly had patients who have felt hopeless and like there’s nothing that’s going to change about the situation’ (participant 38, clinical staff).

‘I have come across over and over and over again people stuck. And I don’t mean just stuck in a room, I mean stuck in a place where there is no hope. There’s no way out, it’s intractable’ (participant 33, commissioner).

Families echoed this sense of futility:

‘There was nothing I could do. You just feel hopeless’ (participant 18, family member).

‘…and I asked three members of the [multidisciplinary team], individually, how do we get her out of LTS? I got three different answers. I just thought, no one understands this. No one knows what is going on and even within this [multidisciplinary team], they don’t know what they’re focusing on’ (participant 76, family member).

Hopelessness permeated every level of the system, reflecting an environment centred on containment rather than rehabilitation. This contravenes the CRPD’s guarantees of equal recognition, liberty and community inclusion (Articles 12, 14, 19) and the CRC’s rights to participation and development (Articles 12, 23).

## Discussion

This study is the first to critically examine long-term segregation (LTS) in mental health hospitals in England through a human rights lens. Drawing on the voices of individuals with lived experience, their families and professionals, the findings demonstrate that LTS is not a therapeutic intervention but a profoundly harmful practice that undermines dignity, autonomy and hope. Participants’ accounts revealed psychological and physical deterioration, emotional trauma and a pervasive loss of self, compounded by institutional cultures of containment and fear. These findings challenge the clinical and ethical legitimacy of LTS and highlight its incompatibility with the principles of rights-based mental healthcare.

### LTS as systemic harm, not therapy

The study findings align with the conclusions of Baroness Hollins,^3^ that all forms of solitary confinement, including LTS, lack therapeutic benefit and may constitute degrading treatment. Across perspectives, LTS emerged as a system of deprivation rather than treatment. Individuals internalised narratives of danger and punishment, families experienced despair and moral distress and professionals described feelings of helplessness within risk-averse cultures. The harms described here – dehumanisation, neglect, fear and hopelessness – mirror violations of CRPD and CRC, both of which guarantee dignity, inclusion and protection from abuse.

Rather than promoting safety or recovery, LTS appears to reproduce and intensify distress. The practice entrenches a cycle of trauma, dependency and risk avoidance that isolates individuals from meaningful human connection. The resulting psychological harm and erosion of trust suggest that LTS not only fails to meet therapeutic aims but actively undermines them.

### International context and reform

The human rights violations identified here are not unique to England. Comparative evidence shows that jurisdictions taking rights-based approaches, such as Norway’s psychiatric reforms, which have significantly reduced coercive practices,^21^ or Australia’s Royal Commission into Violence, Abuse, Neglect and Exploitation of People with Disability,^22^ have achieved measurable reductions in segregation and restraint. These examples reinforce the need for systemic change in the UK to ensure that individuals with learning disabilities and autistic people are protected from coercive and non-therapeutic interventions.

The forthcoming UK Mental Health Bill (2025)^12^ provides an important policy window to embed rights-based principles into law and practice. Our findings offer empirical support for reform by evidencing how LTS contravenes the spirit of the Bill, which seeks to promote autonomy, safety and dignity. Translating these principles into practice will require organisational and cultural change, including the development of viable alternatives to LTS. The HOPE(S) programme represents one such model of reform. Designed to end long-term segregation and improve well-being for those currently subject to it, HOPE(S) provides trauma-informed, rights-based support to individuals, families and clinical teams.

### Policy implications

To prevent the recurrence of harm described in this study, urgent action is required at multiple levels of policy and practice. Table 1 summarises the key recommendations mapped to corresponding CRPD and CRC provisions.

**Table 1:** Policy recommendations to address human rights violations affecting individuals in long-term segregation (LTS)

Area	Recommendation	Relevant rights
Human rights-based care	Ground care planning in dignity, autonomy and participation to prevent dehumanisation, by co-producing plans with individuals (and/or a family member) and evidencing autonomy, consent and regular review.	Convention on the Rights of Persons with Disabilities (CRPD) Articles 3, 12, 19; Convention on the Rights of the Child (CRC) Articles 3, 12
Active monitoring	Monitor well-being (including quality of life and physical health) and intervene early through routine checks and clear triggers for action when isolation or risk narratives emerge.	CRPD Article 25; CRC Article 24
Safeguarding and accountability	Strengthen independent oversight and accountability by expanding unannounced inspections, advocacy access, auditing of restrictive practice and protected whistle-blowing.	CRPD Article 16; CRC Articles 19, 39
Staff training and cultural change	Train staff to uphold rights and support alternatives through mandatory rights-based, trauma-informed approaches and skills to deliver therapeutic and, where appropriate, community-based care.	CRPD Article 19; CRC Article 23
Transition and rehabilitation	Prioritise exit pathways from LTS by requiring time-bound transition plans and holding commissioners and providers accountable for progress into community life.	CRPD Article 14; CRC Article 39

### Limitations and future directions

Although the study included a large overall sample, the number of participants with direct lived experience was low, reflecting ethical considerations around distress and accessibility. Nevertheless, triangulating their experiences with those of families and professionals provides a robust, multi-layered understanding of the systemic nature of harm.

Additionally, the findings and themes may be influenced by self-selection bias, whereby individuals with more strongly negative experiences may have been more inclined to participate, potentially skewing perspectives towards adverse experiences.

Although great strides have been made to provide viable solutions for clinical staff, including piloting of initiatives such as HOPE(S), further research is essential to understand the use and misuse of LTS, as well as how best to support and upskill clinical staffing teams in the use of viable alternatives to restrictive practices. This includes understanding how to prevent individuals from entering LTS resulting from a cycle of restrictive practices; ensuring that their mental and physical health does not deteriorate while in LTS; and identifying and addressing the barriers to transitioning them out of LTS.

LTS causes profound psychological, physical and relational harm and offers no therapeutic benefit. It perpetuates fear, hopelessness and human rights violations, reflecting an institutional culture that prioritises containment over care. These findings strengthen the evidence that LTS should be treated as a ‘never event’ in modern mental health services. Reform must focus on embedding rights-based, trauma-informed approaches, such as HOPE(S), that uphold dignity, safety and recovery for all individuals in care. If the abolition of LTS remains a distant goal, we must urgently embrace openness, transparency and a relentless pursuit of higher standards of care for those subjected to LTS.

### Clinical implications and relevance

LTS is not a therapeutic intervention; it causes psychological deterioration, loss of identity and breaches of human rights.

Staff working within restrictive environments require training and support to adopt rights-based, trauma-informed alternatives such as the HOPE(S) model.

Embedding human rights principles in clinical governance, safeguarding and care-planning is essential to prevent further harm and ensure that LTS becomes a true ‘never event’ in mental health services.

## Supporting information

Fradley and Haines-Delmont supplementary material Fradley and Haines-Delmont supplementary material

## Data Availability

The data-sets generated and/or analysed during the current study are not publicly available due to the sensitive nature of the investigation, and to protect the confidentially of participants, especially those with lived experience, but are available from the corresponding author on reasonable request.

## Supplementary material

The supplementary material is available online at https://doi.org/10.1192/bjo.2026.10996

## References

[1] Quinn A , Cavanagh D , Kilcoyne J , Haines-Delmont A , Ryan S , Lodge K-M , et al. Long-term segregation and seclusion for people with an intellectual disability and/or autism in hospitals: a critique of the current state of affairs: commentary. BMJ Psychiatry 2025; 226: 39–46. 10.1192/bjp.2025.53 40123467

[2] Mencap. Government Must Not Waste Any More Time Following ‘Disturbing’ Findings in Independent Report on Long-Term Segregation in Modern-Day Asylums. Mencap, 2021 (https://www.mencap.org.uk/press-release/government-must-not-waste-any-more-time-following-disturbing-findings-independent).

[3] Department of Health and Social Care. Baroness Hollins Final Report: My Heart Breaks – Solitary Confinement in Hospital Has No Therapeutic Benefit for People with a Learning Disability and Autistic People. Department of Health and Social Care, 2023.

[4] Department of Health. The Mental Health Act 1983: Code of Practice. Department of Health, 2015.

[5] National Institute for Health Care Research. Restrictive Practices in Inpatient Mental Health Settings: A Realist Review. NIHR, 2025.

[6] Care Quality Commission. Review of Restraint, Prolonged Seclusion and Segregation for People with a Mental Health Problem, a Learning Disability or Autism. Care Quality Commission, 2019.

[7] Care Quality Commission. Out of Sight – Who Cares?: Restraint, Segregation and Seclusion Review. Care Quality Commission, 2020.

[8] National Autism Society. CQC Calls For Fundamental Change in the Way Care is Planned, Funded, Delivered and Monitored. National Autism Society, 2020.

[9] United Nations. Universal Declaration of Human Rights. United Nations, 1948 (https://www.un.org/en/about-us/universal-declaration-of-human-rights).

[10] United Nations. Convention on the Rights of Persons with Disabilities (CRPD). United Nations, 2006.

[11] United Nations. Convention on the Rights of the Child. United Nations, 1989.

[12] Department of Health and Social Care. Mental Health Bill. Department of Health and Social Care, 2025.

[13] Tromans SJ , Sawhney I , Odiyoor M , de Villiers J , McCarthy J , Boer H , et al. Long-term segregation and seclusion for people with an intellectual disability and/or autism in hospitals: critique of the current state of affairs. Br J Psychiatry 2025; 226: 39–46. 39629607 10.1192/bjp.2024.211

[14] Fradley K , Rajan DG , Haines-Delmont A. Evaluation of the National HOPE(S) Programme to End Long-Term Segregation (LTS) For Children and Young People, Autistic Adults and/or Adults with a Learning Disability in Inpatient Hospital Settings. Manchester Metropolitan University, 2025 (https://e-space.mmu.ac.uk/640963/).

[15] Kilcoyne J , Angus D. HOPE(S) Clinical Guide to Reduce Long-Term Segregation . NHS Mersey Care, 2023.

[16] Braun V , Clarke V. Thematic Analysis: A Practical Guide. Sage, 2021.

[17] Braun V , Clarke V , Hayfield N , Davey L , Jenkinson E. Doing Reflexive Thematic Analysis. Supporting Research in Counselling and Psychotherapy: Qualitative, Quantitative, and Mixed Methods Research. Springer, 2023.

[18] Finlay L. Negotiating the swamp: the opportunity and challenge of reflexivity in research practice. Qual Res 2002; 2: 209–30.

[19] Finlay L. ‘Outing’ the researcher: the provenance, process, and practice of reflexivity. Qual Health Res 2002; 12: 531–45. 11939252 10.1177/104973202129120052

[20] Department of Health. Care Act 2014. legislation.gov.uk, 2014 (https://www.legislation.gov.uk/ukpga/2014/23/contents).

[21] Husum TL , Finset A , Ruud T. The staff’s emotional reactions to aggressive behavior in mental health services. Nord J Psychiatry 2010; 64: 202–8.

[22] Royal Commission into Violence Abuse, Neglect and Exploitation of People with Disability. Final Report. Royal Commission into Violence Abuse, Neglect and Exploitation of People with Disability, 2023.

